# New Haplotype of *Bartonella* sp. in *Haematopota pluvialis* (Linnaeus, 1758)

**DOI:** 10.3390/pathogens15040417

**Published:** 2026-04-13

**Authors:** Katarzyna Bartosik, Magdalena Świsłocka-Cutter, Joanna Werszko, Anna Aftyka, Klaudia Mária Švirlochová, Dana Zubriková, Bronislava Víchová, Magdalena Raszewska-Famielec, Marek Asman

**Affiliations:** 1 Department of Biology and Parasitology, Chair of Pharmacology and Biology, Faculty of Health Sciences, Medical University of Lublin, Radziwiłłowska 11 St., 20-080 Lublin, Poland; 2 Department of Zoology and Genetics, Faculty of Biology, University of Bialystok, Ciolkowskiego 1J, 15-245 Bialystok, Poland; 3 Department of General Biology and Parasitology, Medical University of Warsaw, Chałubińskiego 5, 02-004 Warsaw, Poland; 4 Department of Anaesthesiological and Intensive Care Nursing, Medical University of Lublin, ul. Chodźki 7, 20-093 Lublin, Poland; 5 Institute of Parasitology, Slovak Academy of Sciences, Hlinkova 3, 040 01 Košice, Slovakia; 6 Department of Epizootiology, Parasitology and Protection of One Health, University of Veterinary Medicine and Pharmacy in Košice, Komenského 73, 041 81 Košice, Slovakia; 7 Faculty of Physical Education and Health, University of Physical Education, 21-500 Biala Podlaska, Poland; 8 Department of Medical and Molecular Biology, Faculty of Medical Sciences in Zabrze, Medical University of Silesia in Katowice, Jordana 19 St., 41-808 Zabrze, Poland

**Keywords:** *Haematopota pluvialis*, *Bartonella*, zoonotic pathogens, Tabanidae bites, *Haematopota* bites, Poland, Europe

## Abstract

*Haematopota pluvialis* is a widely distributed hematophagic insect occurring across Eurasia. This horse fly may be a highly efficient mechanical vector of pathogens, including viruses, bacteria, and protozoa. Furthermore, its painful bites can cause local skin lesions and systemic symptoms. The aim of this study was to determine human exposure to *H. pluvialis* attacks in various types of open space habitats in Eastern Poland and to perform molecular screening of these tabanids for the presence of hematopathogens: *Bartonella* spp. and *Anaplasma phagocytophilum*. Specimens of *H. pluvialis* were collected at three distinct sites in Eastern Poland. The presence of *Bartonella* spp. and *A. phagocytophilum* was investigated using PCR-based methods. In total, 141 *H. pluvialis* females were analyzed. The molecular analysis of the *rpoB* gene fragment yielded one new haplotype of *Bartonella* sp. in 0.7% (1) of all studied samples, which may hypothetically exhibit zoonotic potential. *Anaplasma phagocytophilum* was not detected in the studied material. Moreover, a high level of human and animals exposure to horse fly bites was noted in the studied areas of Eastern Poland. The present results highlight the need for further targeted research on *H. pluvialis* to quantify pathogen prevalence, transmission efficiencies, and conditions facilitating pathogen transmission in natural settings.

## 1. Introduction

**Figure 1:**
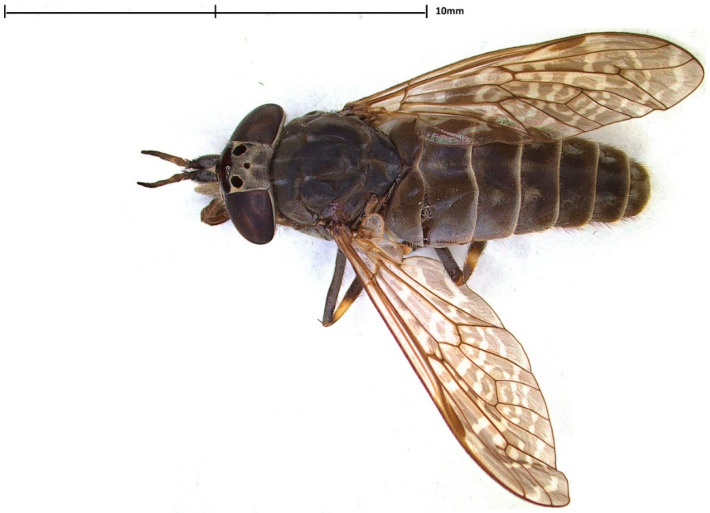
*Haematopota pluvialis* female (Photo by Joanna Werszko).

The Tabanidae family includes over 4000 species globally [1,2,3,4], with *Haematopota pluvialis* (cleg, horse fly) (Figure 1) being one of the most common and widely distributed species across Eurasia [5]. In environmental studies across Europe, including Poland [6], Hungary [7], the Czech Republic [8], Croatia [9], and Spain [10], *H. pluvialis* is frequently identified as the dominant tabanid species, posing a significant nuisance to both animals and humans [10,11]. The occurrence of this hematophagous fly was noted in various biotopes, from lowlands to mountains, most commonly near flowing waters and water reservoirs [4,8]. *Haematopota pluvialis* exhibits a distinct seasonal abundance pattern during late spring and summer, with the peak occurrence varying by geographical location, e.g., early to late June in Croatia [12], the first half of July in Hungary [7], mid to late July in Northern Spain [10], and July in Croatia [9]. Mass occurrence in July and August is observed in Poland ([4,6,13]; own observations). Despite its prevalence, current distribution data remain incomplete, and ecological modeling suggests that its actual range is likely broader than field observations currently indicate [5].

**Figure 2:**
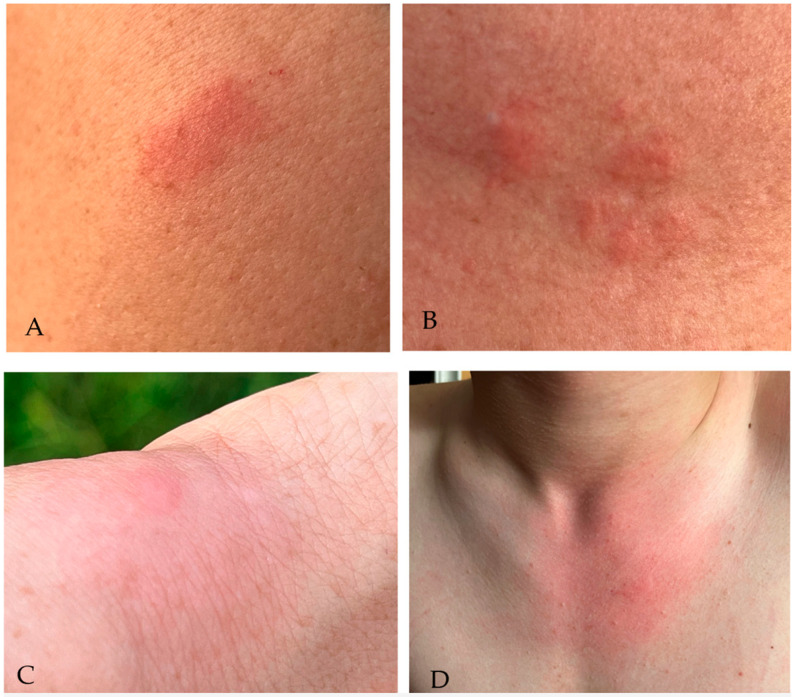
Local skin symptoms following *Haematopota pluvialis* bites. Well-demarcated erythematous-edematous skin lesion located on the arm, observed within the first 24 h following a *H. pluvialis* bite. The lesion presents as a localized area of erythema with associated soft-tissue swelling, clinically reported to be accompanied by pruritus and a burning sensation during the first day (**A**). Multiple confluent erythematous–edematous papules and plaques on an erythematous background located on the anterior chest. An acute urticarial reaction, accompanied by pruritus and burning, was visible on the skin during the first 24 h following the bite (**B**). Localized erythema of the skin overlying the distal forearm with associated soft-tissue swelling around the wrist joint, indicating inflammatory edema. In the central portion, a raised edematous papule corresponding to the bite site is visible, clinically accompanied by intense pruritus and burning during the first 24 h following the bite (**C**); Well-demarcated erythema involving the anterior upper chest and lower neck, measuring approximately 10 cm in diameter, with subtle central edema at the bite site. The lesion is consistent with an acute localized hypersensitivity reaction, reported clinically as being accompanied by intense pruritus and burning sensation within the first 24 h after the insect bite (**D**) (Photos by Katarzyna Bartosik).

The epidemiological significance of *H. pluvialis* is closely related to its feeding behavior. Females are telmophagous (pool feeders) and often require brief, repeated bites from multiple hosts to complete a single blood meal [4,6,14]. The biting activity occurs predominantly during daylight hours, causing notably painful lesions (Figure 2).

This interrupted feeding pattern inherently increases their potential to act as mechanical vectors for various zoonotic pathogens [4,6,14]. Laboratory and in vivo studies have already confirmed that *H. pluvialis* can mechanically transmit viruses such as bovine viral diarrhea virus (BVDV) [15] and lumpy skin disease virus (LSDV) [16]. Furthermore, horse flies have been implicated in the transmission of bacterium *Anaplasma marginale* [17,18] and parasites like *Trypanosoma theileri* [19].

While the role of tabanids in transmitting viruses and certain bovine parasites is documented, their contribution to the circulation of zoonotic bacteria remains a critical research gap.

*Anaplasma phagocytophilum* is an intracellular bacterium, developing mainly in neutrophils. This pathogen is an etiological agent of human granulocytic anaplasmosis and is vectored by ticks. The main symptoms of this zoonosis include fever, headache, myalgia, malaise, thrombocytopenia, and elevated liver enzymes [20]. Moreover, some infections with this bacterium may lead to many adverse complications, including hemolytic and immunological abnormalities [21]. The ability of Tabanidae to transmit *Anaplasma marginale* mechanically between bacteremic and susceptible bovine hosts was demonstrated by Hawkins et al. [17]. These authors also demonstrated that *Tabanus* spp. specimens remain mechanically infective for as long as 2 h after feeding on an acutely infected calf [17]. Roberts and Love observed that splenectomized calves developed anaplasmosis after intravenous application of ground *Tabanus* spp. infected with *A. marginale.* Their study showed that the hemopathogen retains its viability and infectivity within the digestive tract of the studied Tabanidae for at least 2 days [18].

The DNA of the bacterium *A. phagocytophilum*, an etiological factor of granulocytic anaplasmosis in both animals and humans, has been detected in 1.35% (5/27) of *H. pluvialis* collected in NE Poland [22]. Although molecular detection alone does not confirm transmission competence, the presence of pathogen DNA indicates exposure and potential carriage of infectious agents by this species.

*Bartonella* spp. are small, Gram-negative, facultative intracellular bacteria. These pathogens can be transmitted to mammalian hosts by vectors [23]. Currently, over 45 species of *Bartonella* are known, many of which may potentially affect the health of animals and humans [24]. These species primarily infect host erythrocytes and endothelial cells, which may lead to both acute and chronic infections [25]. Blood-feeding arthropods, such as fleas and body lice, are the primary vectors of this bacterium. Other hematophagous arthropods, including clegs, sandflies, biting midges, keds, and ticks, have been documented to carry *Bartonella* spp.; however, their roles in pathogen spread within natural environments are less clearly established [26,27,28,29,30].

Given the wide geographic spread of *H. pluvialis* and its aggressive pursuit of human hosts, there is a need to assess human exposure to *H. pluvialis* attacks in various open-space habitats and perform molecular screening for *Bartonella* spp. and *A. phagocytophilum*. These efforts will contribute to completing the clinical picture of *H. pluvialis* bites, and align with the One Health concept, aiming to better understand the risk horse flies may pose to public health.

## 2. Materials and Methods

### 2.1. Field Studies

**Figure 3:**
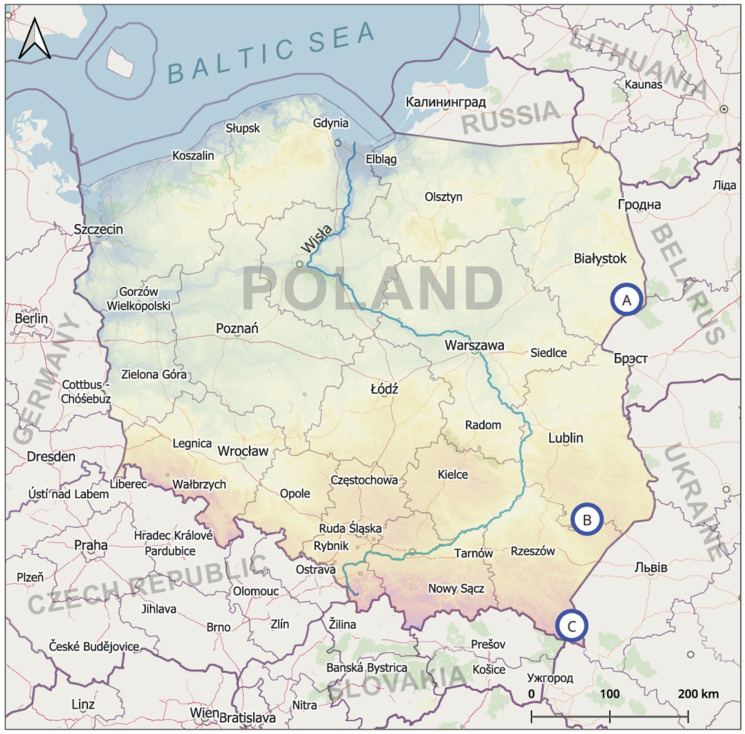
Locations of *Haematopota pluvialis* collection sites A (148 m a.s.l.; 52°45′30.50″ N, 23°36′55.76″ E), B (190 m a.s.l., 50°25′20″ N, 22°56′07″ E), and C (682 m a.s.l., 49°14′50″ N, 22°40′21″ E; 682) in Poland (Source: Marcin Wasilewski, marcinwasilewski.eu on the basis of the OpenStreetMap; Copyright © authors OpenStreetMap).

Specimens of *H. pluvialis* were collected during July and August 2024 using a standard entomological net, as described by Werszko et al. [22,31], at three distinct sites (Figure 3). Confirmation of human exposition was performed by collecting horse flies killed on particular researchers after bite during the field studies. Collection site A was located in a hay meadow adjacent to the forested areas of the Białowieża Forest, at an altitude of 148 m a.s.l. (52°45′30.50″ N, 23°36′55.76″ E). Collection site B was situated on a horse farm in an extensively used pasture in the Szum River valley, Solska Forest, at an altitude of 190 m a.s.l. (50°25′20″ N, 22°56′07″ E), whereas collection site C was located in extensively used meadows in the Western Bieszczady Mountains, at an altitude of 682 m a.s.l. (49°14′50″ N, 22°40′21″ E; 682). Following collection, all specimens were placed in sterile polypropylene tubes, fixed in 70% ethanol, and stored for subsequent morphological and molecular analyses. Species identification and sex determination were performed under a stereoscopic microscope Olympus SZX16 (Olympus, Tokyo, Japan) using a standard taxonomic key by Trojan [4].

### 2.2. Molecular Analyses

Prior to DNA isolation, specimens were washed three times in sterile ultrapure water (A&A Biotechnology, Gdańsk, Poland) and homogenized using tissue grinding tools (EURx, Gdańsk, Poland). The DNA was isolated from 141 single females of *H. pluvialis* using the Kit for genomic DNA purification Xpure™ Cell&Tissue micro (A&A Biotechnology, Gdańsk, Poland) according to the manufacturer’s protocol. Next, DNA concentrations in the isolates were measured spectrophotometrically in the NanoPhotometer PEARL (Implen, Munich, Germany) at the 260/280 nm wavelength. After this stage, the samples were frozen and stored at −20 °C for further molecular analyses.

An endogenous control targeting *H. pluvialis* was included in the molecular analyses as a good molecular practice measure to ensure reproducibility and independent validation of the result by other laboratories. To this end, a fragment of the mitochondrial gene cytochrome c oxidase subunit I (*COI*) was amplified from two randomly selected DNA isolates obtained from studied *H. pluvialis* specimens. A fragment of the *COI* gene was amplified using the universal invertebrate primers LCO1490 (5′–GGTCAACAAATCATAAAGATATTGG–3′) and HCO2198 (5′ TAAACTTCAGGGTGACCAAAAAATCA–3′), as described by Folmer et al. [32]. These primers amplify an approximately 710-bp fragment of the *COI* gene and have been widely applied across diverse metazoan invertebrate taxa. PCR reactions were carried out in a final volume of 25 µL containing: 1× PCR buffer, 2.0–2.5 mM MgCl_2_, 0.2 mM of each dNTP, 0.4 µM of each primer, 1 U of Taq DNA polymerase (EURx, Gdańsk, Poland), and ~20–50 ng of template DNA. Amplification was performed under the following thermal cycling conditions: initial denaturation at 94 °C for 2 min, 35 cycles of denaturation at 95 °C for 30 s, annealing at 53 °C for 60 s, and extension at 72 °C for 60 s, followed by a final extension at 72 °C for 7 min. PCR products were visualized on a 1.5% agarose gel stained with an Midori Green Direct DNA Stain (ABO Sp. z o.o.; Gdańsk; Poland) using a ChemiDoc MP imaging system (Bio-Rad, Hercules, CA, USA). PCR products were purified using the QIAEX II Gel Extraction Kit (Qiagen, Hilden, Germany) according to the manufacturer’s protocol and then sequenced by Genomed (Warsaw, Poland).

*Bartonella* spp. were detected in the insects using the single PCR method. For the detection of pathogen DNA in the studied material, a pair of primers 1400 F(5′ CGCATTGGCTTACTTCGTATG 3′) and 2300R (5′ GTAGACTGATTAGAACGCTG 3′) specific to the *rpoB* gene fragment was used [33]. The reaction mixture contained c.a. 200 ng of matrix DNA, 0.5U (0.1 μL) of DFS-Plus Taq DNA Polymerase (GeneOn, Groß-Rohrheim, Germany), 2.5 μL of the Ammonium-Reaction buffer “complete” with 25 mM MgCl_2_ 10× diluted, 1 μL of 10 mM dNTPs (final concentration 0.25 mM), 1 μL of 5 μM primers 1400F and 2300R, respectively. The samples were made up to a volume of 25 μL with DNase/RNase-free water. DNA of *Bartonella* sp. (GenBank Acc. No. MZ06186) isolated from *Lipoptena fortisetosa* was used as the positive control. For the negative control, ultrapure DNase/RNase-free water was substituted for the template DNA in the PCR reaction mixture. The amplification was performed in a Mastercycler thermocycler (Eppendorf, Hamburg, Germany) under the following conditions: initial denaturation (94 °C for 2 min), proper denaturation (94 °C for 30 s), annealing (53 °C for 30 s), extension (72 °C for 1 min), and final extension (72 °C for 2 min). In total, 35 cycles were performed [33]. *A. phagocytophilum* was detected with the nested PCR method. For the detection of this bacterium in the studied material, two pairs of primers, ge3a (5′ CACATGCAAGTCGAACGGATTATTC 3′) and ge10r (5′ TTCCGTTAAGAAGGATCTAATCTCC 3′), as well as ge9f (5′AACGGATTATTCTTTATAGCTTGCT 3′) and ge2 (5′ GGCAGTATTAAAAGCAGCTCCAGG 3′) specific to 16S rRNA, were used, as proposed by Massung et al. [34]. In addition to c.a. 200 ng of matrix DNA, the reaction mixture contained 0.5 U (0.1 μL) of Taq DNA Polymerase (EURx, Gdańsk, Poland), 2.5 μL of the Pol Buffer C 10 × diluted, containing 15 mM MgCl_2_, 1 μL of 10 mM dNTPs (final concentration 0.25 mM), and 1 μL of 5 μM primers ge3a and ge10r, respectively. The samples were made up to a volume of 25 μL with DNase/RNase-free water. In turn, for re-amplification, 1 µL of amplification product, primers ge9f and ge2, and 18.4 μL of DNase/RNase-free water to a final volume of 25 µL were added. DNA from *A. phagocytophilum* (GenBank Acc. No. GQ450278) isolated from deer was used as the positive control. In the negative control, ultrapure DNAse/RNAse-free water was added to the PCR mix instead of the matrix DNA. The amplification and re-amplification were performed in a Mastercycler thermocycler (Eppendorf, Hamburg, Germany). The amplification conditions were as follows: initial denaturation (94 °C for 2 min), proper denaturation (94 °C for 30 s), annealing (55 °C for 30 s), extension (72 °C for 1 min), and final extension (72 °C for 5 min). In total, 40 cycles were performed. The re-amplification was carried out under the same conditions, but the number of cycles was reduced to 30 [34].

The amplification product was separated electrophoretically in 2% ethidium bromide-stained agarose gels at 100 V for 1 h. Next, the amplicons were visualized under ultraviolet light and photographed in a Vilber Lourmat device (Vilber Lourmat, Collegien, France). The presence of an 825 base pair [bp] amplification product for *Bartonella* spp. as well as 932 bp (first round PCR) and 546 bp (second round PCR) for *A. phagocytophilum* was treated as positive. Next, positive samples (amplicons) of *Bartonella* spp., of size 825 bp, were isolated from the agarose gels and purified using the GeneMATRIX Agarose-OUT DNA Purification Kit (EURx, Gdansk, Poland) according to the manufacturer’s protocol. In turn, sequencing of the positive sample was performed by Genomed (Warsaw, Poland).

### 2.3. Phylogenetic Analyses

Obtained sequences of *COI* gene were edited and assembled using BioEdit v. 7.0.5.3 [35] and compared with reference sequences available in GenBank using the Basic Local Alignment Search Tool (BLAST) to confirm species identity.

The result of sequencing of the *rpoB* gene fragment encoding the RNA polymerase beta subunit was aligned manually using BioEditv.7.0.5.3 [35] and compared with the GenBank references using BLAST (http://www.ncbi.nlm.nih.gov/, accessed on 15 December 2025) to facilitate the identification of *Bartonella* species. The sequence obtained in our analysis was deposited in GenBank (accession no. PX647416). Phylogenetic trees were constructed to assess the relationships between the *rpoB* gene sequence obtained in this study and sequences retrieved from GenBank. The HKY+I+G nucleotide substitution model was identified as the best fitting model according to the Akaike information criterion [36] implemented in jModelTest v.0.1.1 [37]. Using a maximum-likelihood (ML) algorithm in Mega v11.0.13 [38] with 1000 bootstrap replicates to assess the tree node support, we constructed a tree for the *rpoB* gene fragment. We also generated a phylogenetic tree using the Bayesian approach implemented in BEAST v1.7.2 [39]. For our Bayesian analyses, we previously used the Yule process tree [40]. The Bayesian analysis run consisted of an MCMC chain with 30,000,000 iterations sampled every 3000th generation; the first 10% of the iterations were the burn-in. The sampled trees were summarized and annotated in TreeAnnotator v1.7.2 (BEAST software) and visualized in Figtree v1.3.1 (http://tree.bio.ed.ac.uk/software/figtree, accessed on 15 December 2025). The pairwise distance was calculated using the *p-distance* method between the newly obtained sequence of *Bartonella* sp. and sequences with which it was clustered on the phylogenetic trees.

## 3. Results

A total of 141 specimens of *H. pluvialis* from 3 different locations, including 41 females collected in the meadow near the Białowieża Forest (site A), 50 females collected from the pasture on a horse farm in Roztocze (site B), and 50 females collected from the agricultural area in the Bieszczady Mountains (site C), were analyzed with molecular methods. These numbers of insects were caught in entomological nets during two 45-min collections conducted at each of the three research sites. The number of *H. pluvialis* females killed/captured on humans during field studies was 74, including 18 in habitat A (7 and 11 per person), 31 in habitat B (11 and 20 per person), and 25 in habitat C (9 and 16 per person). High exposition to the bites was the reason why field studies were limited in time. In total, *Bartonella* sp. was detected in 1/141 (0.7%) of all studied samples. This bacterium was detected only in one female of *H. pluvialis* collected in the Roztocze area (1/50 (2%) of specimens collected in this habitat). *Anaplasma phagocytophilum* was not detected in the studied material.

### Phylogenetic Analyses

Two *COI* haplotypes revealed in *H. pluvialis* derived DNA isolates, each 707 bp in length, were deposited in GenBank under accession numbers PZ049887 and PZ049888. The two obtained haplotypes differed by 10 transitions and one transversion. Haplotype H1 (GenBank accession no PZ049887) showed 99.43% sequence identity to a previously reported *H. pluvialis* haplotype (GenBank accession no. MT584146; unpublished data) and 99.01% identity to haplotype of *H. pluvialis* published by Wiegmann et al. [41] (GenBank accession no. KC192969). The our H2 haplotype (GenBank accession no. PZ049888) showed 99.01% and 98.30% sequence identity to haplotypes MT584146 and KC192969, respectively.

**Figure 4:**
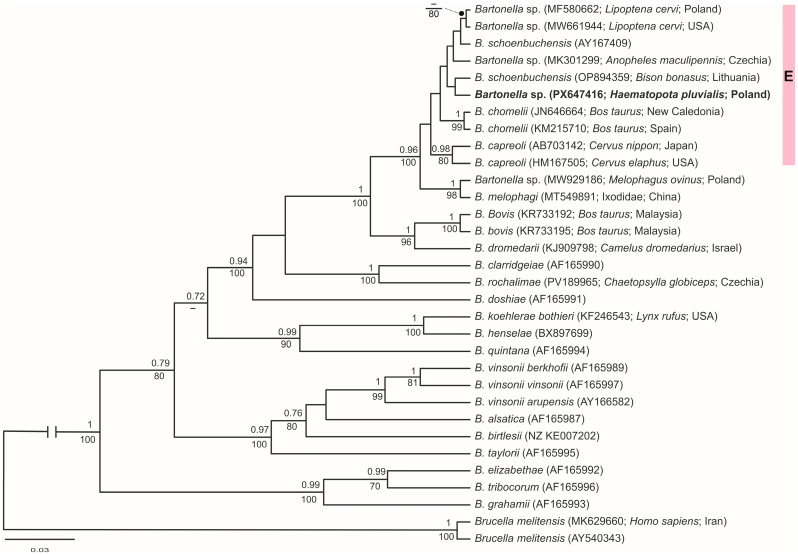
Bayesian phylogenetic tree inferred using the HKY+I+G sequence evolution model, showing the phylogenetic relationships between the *rpoB* gene fragment for the RNA polymerase beta subunit sequence of *Bartonella* sp. obtained in this study and reference haplotypes retrieved from GenBank. Numbers above the nodes indicate posterior probabilities estimated by Bayesian inference (MrBayes), whereas numbers below the nodes represent node support (%) based on 1000 bootstrap replicates from the maximum-likelihood analysis. The *Bartonella* sp. haplotype found in this study is shown in bold. The haplotype identified in this study belongs to phylogenetic lineage E, as described by Sato et al. [45].

The molecular analysis of the 816-bp fragment of the *rpoB* gene encoding the RNA polymerase beta subunit revealed a novel *Bartonella* sp. haplotype (GenBank accession no. PX647416). In both maximum-likelihood (ML) and Bayesian phylogenetic analyses, this haplotype clustered with sequences of *Bartonella schoenbuchensis* described from Lithuania (OP894359, [42]; pairwise distance: 0.007) and sequences previously reported from Poland (MF580662, [29]; pairwise distance: 0.009), the Czech Republic (MK301299, [43]; pairwise distance: 0.005), and the USA (MW661944, [44]; pairwise distance: 0.014), as well as *B*. *schoenbuchensis* sequences from France (Figure 4). These haplotypes, together with sequences of *B. capreoli,* belong to phylogenetic lineage E, as described by Sato et al. [45].

## 4. Discussion

Although *H. pluvialis* is not the primary biological vector for most pathogens endemic to Europe, its ecological and behavioral characteristics enable it to contribute to the mechanical transmission of pathogens in agricultural landscapes. As a mechanical vector, it serves as a passive carrier of infectious agents from infected hosts or contaminated sources to susceptible hosts, without pathogen development, multiplication, or specific interaction within the vector. In arthropods, the mechanical transmission of pathogens typically occurs through external body parts, such as mouthparts, or by contact with contaminated surfaces, followed by deposition on the food, tissues, or skin of another host [46].

Due to their biological features and morphology, tabanids are considered one of the most significant hematophagous Diptera implicated in the mechanical transmission of infectious agents [47,48,49,50,51]. Belonging to the family Tabanidae, *H. pluvialis* is a highly mobile, intermittent feeder, characterized by a frequent and rapid feeding pattern [3,4,50]. This habit of interrupted feeding, combined with a broad host range, increases contact with reservoirs of pathogens and enables tabanids to mechanically transmit the etiological factors of several diseases, e.g., anthrax, tularemia, anaplasmosis, and trypanosomiasis caused by *Trypanosoma vivax* [3,4,50,51]. However, it should be stated that certain Tabanidae species, including *H. pluvialis*, also serve as vectors for the *T. theileri* complex which belong to the stercorarian section of trypanosomatids [19]. The large size of the mouthparts combined with the volume of blood collected favor adhesion and carriage of infectious agents. The short time intervals between subsequent feedings increase the probability that pathogens remain viable on the vector’s body long enough to reach a susceptible host [4,52]. Finally, the broad ecological niche and widespread distribution of this species across varied habitats, numerous vector populations, and exposure to multiple bites combined with the long duration of its diurnal activity enhance its potential for contact with diverse host species, including reservoir populations [4,5,8].

Molecular surveys indicate that keds and other hematophagous flies frequently harbor *Bartonella* spp. DNA, often corresponding to host-associated genotypes [26,28,29,30]. The high prevalence in keds, the evidence for transstadial and possible vertical transmission, and the identification of distinct *Bartonella* spp. lineages support the hypothesis that, in addition to fleas, body lice, flies, and ticks, other hematophagus arthropods may be involved in the circulation of these bacteria in nature. However, determining vector competence versus passive carriage requires further investigation in controlled conditions. In the present study, *Bartonella* sp. was detected in *H. pluvialis* collected from a pasture where a herd of primitive Polish horses constantly graze. To the best of our knowledge, this represents the first record of *Bartonella* sp. in clegs. The phylogenetic analysis based on the *rpoB* gene revealed a novel haplotype of *Bartonella* sp. belonging to the E phylogenetic lineage of this species [45].

The smallest pairwise genetic distances were found in comparisons of the newly obtained *Bartonella* sp. sequence with the sequence of *Bartonella* sp. (MK301299; 0.5%) (not published) and *B*. *schoenbuchensis* (OP894359; 0.7%) isolated from *Bison bonasus* spleens in Lithuania [42]. Considering the phylogenetic placement of the detected *Bartonella* sp. haplotype within lineage E, we hypothesize that this variant may possess zoonotic potential. Additionally, it is presumed that the source of this novel haplotype was a blood meal obtained from one of the relatively common Cervidae species inhabiting the study area.

Sequence analysis of the *rpoB* gene has been demonstrated to provide high discriminatory power for bacterial identification [33]. In the genus *Bartonella*, *rpoB* has been widely applied as a reliable and informative molecular marker for detection and preliminary species identification. However, robust species delineation and phylogenetic classification within this genus typically rely on multilocus sequence analysis (MLSA) rather than single-locus approaches [53]. Single-gene analyses may not fully resolve closely related or recently diverged taxa, potentially limiting taxonomic resolution. Housekeeping genes such as *gltA*, *ftsZ*, and *groEL* have been extensively used to enhance phylogenetic accuracy and taxonomic stability [54]. Therefore, although *rpoB* was considered sufficient for initial screening and detection in the present pilot study, particularly given that all amplicons were sequencing-confirmed, we acknowledge that comprehensive taxonomic characterization requires a multilocus framework. Future investigations will expand the molecular panel to include additional genetic loci, enabling more robust species identification, improved phylogenetic reconstruction, and deeper insight into the evolutionary relationships and host-associated diversity of the *Bartonella* strains identified in this study.

Our molecular findings highlight the potential role of *H. pluvialis* in the mechanical transmission or incidental carriage of zoonotic *Bartonella* sp., warranting investigation into its epidemiological significance. In central Italy, in areas with a high abundance of hematophagous arthropods, seroprevalence of antibodies against *Bartonella henselae* was detected in 58.4% (45/77) of horses. Among the seropositive animals, only 26.6% (12/45) were PCR-positive for *Bartonella* sp., confirming the possibility of *Bartonella* sp. infection in horses [55]. Seroconversion and bacteremia in horses infected with *B. henselae* via intradermal inoculation were previously demonstrated by Palmero et al. [56]. *Bartonella henselae*-inoculated horses developed local skin reactions, regional lymphadenopathy, and limb edema, which represent clinical signs of *B. henselae* infection [56]. While chronic bacteremia in horses is unlikely, the role of these animals in the circulation of *Bartonella* sp. in nature requires further investigation. Although direct experimental transmission data remain limited, the ecology and behavior of *Haematopota* spp. support their putative role as mechanical vectors. Many horseflies demonstrate notable dispersal capacities (up to ~200 m from release points), facilitating potential spread of pathogens within herds or across adjacent animal populations [57].

Trojan, citing research by Łutta (1970), described the distribution of blind flies on the horse’s body during blood sampling. In the case of *Haematopota* spp., bites were located mainly on the head (39%), neck (39.3%), trunk (8.4%), abdomen (7.2%), shoulder and upper arm (4.8%), forelegs (3.6%), hind legs (2.5%), and flanks (2.4%) [4]. According to Yoshimeki, when choosing a feeding site, an important factor for Tabanidae seems to be the temperature and humidity of individual parts of the host’s body, as opposed to the thickness of the skin or hair barrier [58]. In turn, analogous studies conducted with the participation of human hosts showed that the most common feeding sites of *H. pluvialis* were the head (61.1%) and neck (31.9%), while bites on the upper leg, chest, upper arm, and forearm were observed in only 0.7–2.7% of studied subjects. The authors speculated that the observed pattern of bite distribution may be caused by the insects being attracted by CO_2_ and its increased concentration in the respiratory tract area [11]. In this study, a similar distribution of *H. pluvialis* bites on humans was observed while the researchers were exposed to the bites. Although the lesions shown in Figure 2 are located outside the head area, most horse fly bites were observed on the head, mainly around the face, and the resulting skin lesions resembled those shown in Figure 2A,B,D.

During blood-feeding, the parasitic female of *H. pluvialis* uses its mouthparts to lacerate the skin and subcutaneous capillaries, forming a small “pool” of blood, lymph, and saliva from which it feeds, which is the essence of the telmophagic feeding pattern [4]. Consequently, bites elicit characteristic cutaneous and immunologic responses in the host, attributed to mechanical tissue injury, action of salivary bioactive compounds, and host immune response. The robust, serrated Tabanidae mouthparts cut into the skin, causing painful wounds and local inflammation [4]. The strong activity of hyaluronidase combined with anticlotting and antithrombin factors present in Tabanidae salivary gland extracts probably facilitate the spread of other bioactive salivary components contributing to the formation of the feeding hematoma [59,60]. The dermatologic manifestation of a horsefly bite typically includes a wheal-and-flare reaction with erythema, edema, and pruritus at the site of the bite. These local reactions reflect acute inflammatory processes and can persist for hours to days. In clinical reports of Tabanidae bites, lesions have been described as erythematous plaques often with a prominent central point corresponding to the bite site, frequently accompanied by localized pain and a burning sensation. Local inflammation and tissue injury from the mechanical incision combined with histamine release and vascular responses contribute to this reaction ([61,62], own observations). During fieldwork conducted as part of this study, cases of *H. pluvialis* bites on humans associated with erythema, swelling, severe pruritus, and a burning sensation have been documented (Figure 2A–D).

Beyond local reactions, horse fly bites can induce allergic hypersensitivity mediated by host immune responses to salivary proteins. Cases documented in the literature report IgE-mediated allergic reactions, e.g., generalized urticaria, angioedema, bronchial constriction, and anaphylaxy, specifically caused by *H. pluvialis* bites [63,64,65]. Freye and Litwin pointed to a higher risk of anaphylactic reactions to Diptera bites in subjects sensitized to hymenoptera [64]. Quercia et al. presented two cases of a serious 3–4 degree-type Mueller classification systemic reaction after a horsefly bite. Based on clinical observations and co-sensitization patterns, cross-reactivity between horse flies and Hymenoptera in a form of wasp-horsefly syndrome has been suspected with hyaluronidase as the cross allergen [65].

Hemmer et al. demonstrated IgE-mediated anaphylaxis to *Chrysops* spp. salivary 69 kd protein—the first allergen identified from tabanids. The researchers indicate that, because *Haematopota* spp. is the most important Tabanidae genus for humans in the northern hemisphere, allergy testing should be performed with *Haematopota* spp. extracts rather than with *Chrysops* spp. or *Tabanus* spp., which are currently used in commercial extracts. The authors also conclude that allergy to tabanids may be more common than previously thought, as underreporting may result from limited diagnostic resources and insufficient medical awareness [66].

The mechanical disruption of the skin barrier and persistent inflammation can predispose the bite site to secondary infections by both direct mechanical pathogen transmission through the bite or in the case of inadequate care.

## 5. Conclusions

This study reports the first detection of *Bartonella* sp. in *H. pluvialis*. However, the low prevalence (0.7%) indicates that the available data are insufficient to conclusively determine the epidemiological significance of this horse fly in pathogen transmission within natural environments. Field observations at all study sites demonstrated substantial human and animal exposure to horse fly bites, highlighting the insect’s aggressive host-seeking behavior across various habitats. Considering the medical and veterinary importance of *H. pluvialis* as a potential mechanical vector and the high exposure of hosts to multiple and repeated bites, molecular screening for the presence of infectious agents should be continued along with research on repellents that can provide effective protection against *H. pluvialis* bites. In addition to molecular screening, further investigations should incorporate in vitro cultivation protocols, particularly for pathogens such as *Bartonella* spp., to substantiate the presence of viable, infectious bacteria rather than relying solely on genomic detection.

## Data Availability

Data is contained within the article.
